# L-alanine supplementation in Pompe disease (IOPD): a potential therapeutic implementation for patients on ERT? A case report

**DOI:** 10.1186/s13052-022-01249-y

**Published:** 2022-03-28

**Authors:** Valentina Rovelli, Juri Zuvadelli, Marta Piotto, Andrea Scopari, Alice Re Dionigi, Vittoria Ercoli, Sabrina Paci, Graziella Cefalo, Elisabetta Salvatici, Giuseppe Banderali

**Affiliations:** Clinical Department of Pediatrics, San Paolo Hospital, ASST Santi Paolo E Carlo, University of Milan, Via Antonio di Rudinì 8, 20142 Milan, Italy

**Keywords:** Pompe Disease, Enzyme replacement therapy, L-alanine, Myopathy, Body composition

## Abstract

**Background:**

Pompe disease (PD) is a disorder of glycogen metabolism conditioning a progressive and life conditioning myopathy. Enzyme replacement therapy (ERT) is currently the best treatment option for PD, but is not resolutive. While other potential therapeutic approaches have been reported before, these have never been tried as co- treatments. L-alanine oral supplementation (LAOS) has been proven to reduce muscle breakdown: we hereby report the first case of supplementation on a PD patient on ERT.

**Case presentation:**

F. is a 9 y.o. infantile onset Pompe Disease (IOPD) girl ERT-treated since age 1 developing a progressive myopathy. We started her on LAOS and performed assessments at baseline, 6 and 9 months. At baseline, F.’s weight, height and BMI were within normal ranges, while body composition showed low fat mass -FM and high resting energy expenditure—REE levels. After LAOS, a progressive FM increase and REE reduction could be observed both at 6 and 9 months.

**Conclusions:**

ERT is not curative for PD patients thus additional treatments could be considered to improve outcomes. Our patient showed physical signs of inability to accumulate energy when exclusively on ERT, while FM increase and REE reduction occurred when supplemented with LAOS, likely reflecting anabolic pathways’ implementation. This is the first case reporting potential LAOS benefits in PD-on ERT patients. Longitudinal case control studies are yet needed to evaluate possible efficacy of combined LAOS And ERT treatment in PD patients.

## Background

Pompe disease (PD), or glycogen storage disorder type II (GSD II; OMIM 232,300), is an inherited lysosomal disorder due to the deficiency of the acid a-glucosidase enzyme (GAA, EC 3.2.1.20) causing multisystemic glycogen accumulation [1]. The latter leads to variable associated phenotypes: from severe rapidly progressive forms, usually presenting with hypertrophic cardiomyopathy, to slowly progressive later-onset forms with typical muscular weakness, especially in the legs and the trunk, that can occur from early childhood to late adulthood and typically without cardiac involvement. Numerous causing variants in the GAA gene have been described to date, mostly homozygous or in compound heterozygosity, defining a continuous spectrum of clinical presentations [2] for which it is difficult to establish a strict genotype/phenotype correlation. Enzyme-replacement therapy (ERT) with recombinant human GAA (rhGAA) [3, 4] is currently the main treatment available for patients, as it seems capable of highly reverting cardiac muscle involvement and extending life expectancy in infantile patients; still, it seems lacking in treating residual symptoms, e.g. skeletal muscle involvement [5]. Before ERT, many other potential treatment strategies have been attempted, such as dietary interventions and oral supplementions [6–9], as inactivity and/or inadequate/excess food intake were shown to maybe play a crucial role accelerating disease progression [10]. Among those tried, L-alanine supplementation has been shown to reduce protein degradation and muscle breakdown, being a gluconeogenic amino acid capable of decreasing branched-chain AA catabolism even with relatively short period treatment-trials [11]. This is also cheap, palatable and well tolerated by patients, with minimal energy burden [12]. Reported trials have been in ERT naïve patients to date, while supplementation has never been tried in on-ERT PD patients and potential outcomes are yet to be described.

## Case presentation

F. is a 9 yo IOPD affected girl born at term after an uneventful pregnancy and neonatal period. Unremarkable family history. At age 1 m.o., due to the incidental finding of heart murmur, heart US was performed and resulted normal. At age 7 m.o. she was admitted for pneumonia and hypertransaminasemia (AST 168 U/L, n.v 14–36 U/L, ALT 102 U/L, n.v. 9–52 U/L). Chest X-ray showed left pulmonary thickening with no cardiomegaly. At that time, there was no history of feeding problems, failure to thrive, hypotonia or muscle weakness. Pneumonia remitted after a standard antibiotic therapy, but hypertransaminasemia and altered levels of other serum muscle breakdown enzymes persisted at several follow ups (AST up to 388 U/L, ALT up to 164 U/L, CPK up to 942 U/L, n.v. 30-135U/L, LDH up to 4851 U/l, n.v. 313–618 U/L). Most frequent causes of such enzymes alterations were ruled out throughout follow up until signs of left ventricular hypertrophy could be highlighted at the ECG, which was followed by heart US confirming mild biventricular hypertrophy. F. was then again admitted at age 1 yo. while developing mild limbs tongue protrusion, hypotonia and hyporeflexia, though still preserving axial tone suggesting a peripheral cause such as a myopathy. Abdominal US, EEG and brain MRI were performed and resulted normal. Diagnostic suspicion of PD emerged, thus alfa-glucosidase activity dosage was performed resulting absent in fibroblasts and low in leukocytes (0.22 U.M, normal value > 0.35 U.M.). Molecular GAA gene analysis subsequently confirmed compound heterozygosis for 2 previously described variants (c.1655 T > C, p.Leu552Pro described as severe [13]; c.1927G > A, p.Gly643Arg described as less severe and associated with Late-Onset PD [14]). After CRIM assessment (resulted positive), ERT was started immediately (Myozyme®, iv, 20 mg/kg/2 weeks) and cardiac hypertrophy reverted and was complete already after 8 months of ERT initiation. Antibody status was monitored and seroconversion was gained after 6 months of therapy (maximum reached titer to date is 1600, stable in the last years; no immunomodulatory therapy has been started to date). Nutritional counselling ensured a high protein diet (> 25% of energy requirements as suggested by literature for PD patients [6, 7]) and adequate intakes of other macro and micronutrients according to LARN (Nutrient and energy reference intake levels for the Italian population) [15], with a 3 days dietary food record repeatedly performed for monitoring purposes [16]. F. always showed a linear growth-rate with anthropometric indices comparable to healthy population. Despite early beginning of ERT, F. is developing slowly progressive myopathy conditioning walking difficulties, moderate respiratory impairment and oropharyngeal dysphagia. Muscle MRI was performed, demonstrating bilateral gluteal muscle hypotrophy with preserved trophism of the thigh and leg muscles in the absence of significant signs of fibroadipose infiltration; also hyperintensity of the anterior lodge muscles was found (especially the quadriceps).

We tried to investigate our patient for testing possible effects of L-alanine oral supplementation (LAOS) during ERT. Anthropometric measures, including weight, height and BMI were evaluated and relative z-scores generated using the WHO 2007 growth charts [17]. Body composition was assessed using air displacement plethysmography (BOD-POD®), providing fat mass (FM), fat free mass (FFM) and relative percentages according to McCarthy 2006 percentiles [18]. Indirect calorimetry [19] (Q-NRG, Cosmed®) was used to measure resting energy expenditure (REE) under standardized conditions. Prior to study start, dietary habits were analyzed with a 3-days-food dietary record. The patient was then requested not to change dietary habits or exercise pattern throughout the study. Plasmatic alanine levels, alanine/lysine ratio, muscle enzymes, renal function and nutritional indices were included in the study protocol as monitored indices. L-alanine was administered as powder, mixed into a drink or creamy food (mainly milk or yogurt), starting at age 8 yo and 6 mo, with a starting dose of 0.5 g/kg/day TTD for a total of 15 g/day. Dosage was maintained unchanged for 6 months total (T1), then increased to 0.6 g/kg/day (total 18 g/day) for other 3 months (T2, 9 months after LAOS start). Patient was instructed to have an usual overnight fasting for biochemical examinations to be performed during the morning, in a fasting state and a thermo-neutral environment with the patient supine and awake [19]. Assessments took place at baseline (T0), at 6 (T1) and 9 months (T2).

**Fig. 1:**
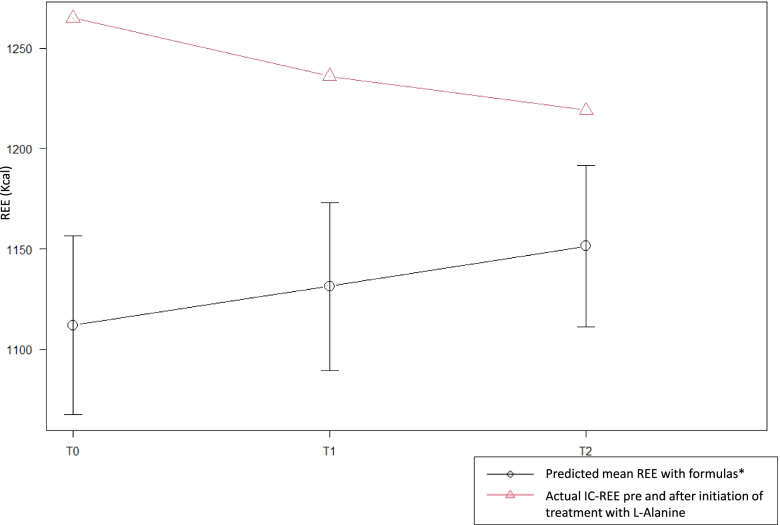
Time-REE correlations with LAOS, patient on treatment vs. expected patient’s REE without intervention. Shapiro–Wilk normality test and repeated-Measures ANOVA were performed (with statistical significance *p*-value set at < 0.05) for studying the variables at each studied time with data grouped into two categories. First group contained the variables of basal metabolism repeated at 3 times of our patient (red line). Second group contained instead the expected basal metabolism variables without intervention, obtained with three different formulas* (Harris Benedict-REE; Schofield-REE; WHO-REE) at same three different times and based on patient’s parameters (black line). A statistically significant association can be found between intervention and time with the basal metabolism of our patient (*p*-value 0.004)

At baseline, F.’s weight, height and BMI were within normal ranges, while BC demonstrated low FM (9.9%, < 2°pc) and high REE levels (112%, 1265 kcal/day vs. predicted WHO rates = 1133 kcal/day). These results, especially those related to FM, were in line with MRI previous findings (high intensity on T2WIs might suggest fatty infiltration or edema/inflammation and STIR can differentiate between them: suppressing fat signals, inflammation/edema was confirmed as major possible causal factor, thus low FM was in line with findings). 3-days-food dietary record analysis confirmed a diet higher in proteins (≥ 3.0 g/kg/day), corresponding to 20–25% of total daily energy intakes. This remained unchanged throughout all study period and still is ongoing. During the study period, the patient was compliant with LAOS treatment protocol and could complete all assessments due, no complications occurred. A progressive FM increase (9.9% 11.6% 13.4%) could be observed over the study period (Table 1) and IC-REE, remarkably high at baseline [20], decreased with a variation of 10% at T2 (44.7 to 40 kcal/kg/day). These modifications were significant: a statistically significant association was found between LAOS and time (*p*-value 0.004) (Fig. 1). IC-REE, expressed as Kcal/day, decreased with a variation of 3.6% compared to an expected increased (+ 2.1; + 4.2; + 4.3) according to Harris Benedict, Schofield and WHO respectively (Table 2). Changes in biochemical indices were unremarkable over study period and indices of muscle damage remained substantially unchanged, including ALT, AST, CPK, LDH, transthyretin, albumin, Ala/Lys ratio and lactate. No increases above considered normal values in Alanine blood concentrations could be observed during study period. On a patient’s perspective, even if difficult to objectify, it’s good to report that F. is now reducing the need of walking supports, she’s capable of standing and walking alone and can now sleep without any pillows.

**Table 1:** Anthropometric measures and evaluated indices of body composition before and during LAOS

	T0	T1	T2
Weight (kg)	28,3	29,4	30,5
Weight z-score (SD) §	0,36	0,15	0,17
Height (cm)	137,2	139,7	141,4
Height z-score(SD) §	1,27	1,03	1,03
BMI (kg/m2)	15,0	15,1	15,2
BMI z-score (SD)§	-0,49	-0,62	-0,58
FM %	9,9	11,6	13,4
FFM%	90,1	88,4	86,6
FM Kg	2,8	3,4	4,1
FFM Kg	25,5	26,0	26,4

T0: baseline pre-LAOS; T1: after 6 months of LAOS start at 15 g/day; T2: after 3 months of increasing LAOS dosage (18 g/day) for a total of 9 months supplementation. Notes: § WHO 2007 Growth Charts

**Table 2:** REE parameters analysis obtained from indirect calorimetry (IC) compared to prediction with commonly used equations

		T0	T1	T2	Percentage variation (T0-T2)
IC-REE (kcal/day)		1265	1236	1219	-3.6
^*£*^*Harris Benedict-REE( Kcal/day)*		1142	1153	1166	+ 2.1
^*§*^*Schofield-REE (Kcal/day)*		1061	1083	1106	+ 4.2
^*¤*^*WHO-REE (Kcal/day)*		1133	1158	1182	+ 4.3
IC REE/predictive equation (%)	*Harris Benedict*	11.1	10.7	10.5	-5.4
	*Schofield*	11.9	11.4	11.0	-7.6
	*WHO*	11.2	10.7	10.3	-8.0

T0: baseline pre-LAOS; T1: after 6 months of LAOS start at 15 g/day; T2: after 3 months of increasing LAOS dosage (18 g/day) for a total of 9 months supplementation. Notes: IC-REE: REE measured by indirect calorimetry; %IC-REE/predictive equation: ratio of IC-REE to predicted equations; *£ Harris Benedict-REE: Harris JA, Benedict FG. A biometric study of basal metabolism in man. Washington: Carnegie Institute of Washington;1919. § Schofield-REE: Schofield WN. Predicting basal metabolic rate, new standards and review of previous work. Hum Nutr Clin Nutr 1985;39:5–41. ¤ WHO-REE: World Health Organization. Energy and protein requirements, Report of a joint FAO/WHO/UNU expert consultation. Geneva: World Health Organization;1985*

## Discussion and conclusions

Pompe disease is known to cause a severe progressive myopathy and other harmful clinical issues. At present, ERT has been proven to be successful delaying and halting progression of the disease, however myopathy remains present and can progress thus a critical evaluation of other potential beneficial therapies is still needed to enhance the approach to PD. Myopathy’s etiopathogenesis in PD is yet to be completely understood but the progressive glycogen accumulation in lysosomes and related membranes damage seems to be responsible for the muscle contractile units’ impairment. Nonetheless, there is recent evidence on a possible defective pathway along autophagic ways that could play a major role in muscle damage [21]. Although little is yet known about these processes, it’s unquestionable that muscle atrophy occurs when the balance between protein synthesis and degradation is unbalanced and shifted in favor to the latter [22]. Several approaches have been studied over years to obtain a slowdown in protein breakdown and consequent muscle damage. Among those, diets higher in proteins and a potential L-Alanine supplementation seem to be the most favorable, the first ensuring an increased pool of amino acids which could make up for the proteolysis and slow down the muscular damage progression [6, 23], the latter being a gluconeogenetic aminoacid thus enhancing glucose availability and sparing the branched-chain aminoacids leading to reduced leucine oxidation (an indirect index of protein breakdown) [11]. Such interventions have been tried to date only before ERT advent and demonstrated a REE reduction [12] and increase in muscle mass [24] defining a possible valid approach to reduce proteolysis. Despite this, after ERT approval this approach has never been tested again and focus has shifted on how to avoid FM reduction as marker of muscle substitution. Even if this could be relevant for clinical purposes, there is also evidence in literature on how possibly an overmuch FM reduction could also be disadvantageous: healthy or underweight individuals with reduced FM seem to be at higher risk of frequent events of chronic obstructive pulmonary disease [25]. These observations led us to investigate more, attempting a L-Alanine oral supplementation in a IOPD patient on ERT. Before LAOS, our patient demonstrated a normal BMI with low FM and elevated REE, reflecting the potential patient’s inability to accumulate energy and the predominance of catabolic over anabolic processes as eventual index of increased protein breakdown. Over study period, a positive change in body composition with increasing FM percentages and a reduction of REE could be observed. Although BMI remained substantially unchanged, the meaningful reduction of REE can reflect the improvement in body composition and energy balance with implementation of anabolic pathways. Body composition standards for PD patients are not known, also considering the wide range of disease clinical presentation, and there are no specific-to-disease growth charts available. We then used general population as targeted comparison, aiming to reach similar FM/FFM distribution to optimize metabolic functioning. There is still lack of evidence about the translation of improved body composition in functional outcomes and the role of L-alanine supplementation in clinical improvements. However, our results suggest that LAOS may improve body composition and ameliorate resting metabolism in PD patients even on-ERT thus should be implemented in treatment protocols. Longer time-periods of LAOS and studies on larger numbers of patients are needed to confirm and ensure our results and to fix optimal dosage to be used, but we expect that they could highlight even more significant clinical changes.

## Data Availability

All data are stored in the San Paolo Hospital of Milan (Italy), Clinical Department of Pediatrics. The datasets used and/or analysed during the current study are available from the corresponding author on reasonable request.

## Abbreviations

PD: Pompe disease
ERT: Enzyme replacement therapy
LAOS: L-alanine oral supplementation
BMI: Body mass index
FM: Fat mass
FFM: Fat free mass
REE: Resting energy expenditure
BC: Body composition
GSD: Glycogen storage disorder
GAA: Acid a-glucosidase enzyme
US: Ultrasonography
ECG: Electrocardiographic
MRI: Magnetic resonance imaging
CRIM: Cross Reactive Immunological Material
TTD: Three times a day
WHO: World Health Organization
